# An autoinflammatory RIG-I variant causing Singleton-Merten syndrome associates with small non-coding Y-RNAs

**DOI:** 10.1093/discim/kyag013

**Published:** 2026-07-01

**Authors:** Benjamin J Thompson, Christ C P Leemans, Dennis Gravekamp, Amarise-Jourmaine M H Silie, Jorn E Stok, Jasper W de Wolf, Erik B van den Akker, Frank J T Staal, Hailiang Mei, Annemarthe G van der Veen

**Affiliations:** Department of Immunology, Leiden University Medical Center, Leiden, The Netherlands; Department of Immunology, Leiden University Medical Center, Leiden, The Netherlands; Department of Immunology, Leiden University Medical Center, Leiden, The Netherlands; Department of Immunology, Leiden University Medical Center, Leiden, The Netherlands; Department of Immunology, Leiden University Medical Center, Leiden, The Netherlands; Department of Immunology, Leiden University Medical Center, Leiden, The Netherlands; Department of Immunology, Leiden University Medical Center, Leiden, The Netherlands; Department of Immunology, Leiden University Medical Center, Leiden, The Netherlands; Department of Biomedical Data Sciences, Sequencing Analysis Support Core, Leiden University Medical Center, Leiden, The Netherlands; Department of Immunology, Leiden University Medical Center, Leiden, The Netherlands

**Keywords:** type I interferonopathies, Singleton-Merten syndrome, RIG-I-like receptors, type I interferon response, autoinflammation

## Abstract

**Introduction:**

The RNA sensor retinoic acid-inducible gene I (RIG-I) performs a critical role in surveying the cytoplasm for the presence of viral nucleic acids and initiating the downstream anti-viral type I interferon pathway. Through recognition of specific features, such as the presence of a 5′ tri/diphosphate motif or highly structured base-paired regions, RIG-I effectively differentiates between RNA of viral- and self-origin. In Singleton-Merten syndrome (SMS), gain-of-function variants in RIG-I lead to a breakdown in this surveillance system and results in aberrant sensing of self-RNAs and deleterious upregulation of type I interferons. The identity of the self-RNAs binding to gain-of-function RIG-I mutants has remained elusive and their elucidation would provide a greater understanding of the aetiology of SMS.

**Methods:**

Here we used an infrared individual-nucleotide resolution UV-crosslinking and immunoprecipitation (irCLIP) approach to determine the RNA profile bound to a previously characterized ATPase-deficient SMS variant, RIG-I^C268F^.

**Results:**

irCLIP identified a broad array of self-RNAs, primarily those transcribed by RNA polymerase III, that were bound to RIG-I. Subsequent native RNA immunoprecipitation confirmed a prominent and specific interaction between RIG-I^C268F^ and Y-RNAs, a family of four structurally similar non-coding RNAs.

**Conclusion:**

Manipulation of Y-RNAs alone by targeting either Y-RNA transcripts or Y-RNA stabilizing proteins was insufficient to negate RIG-I induced interferon responses, hinting at a broader profile of RNA polymerase III-derived RNAs being influential in driving the sterile activation of gain-of-function RIG-I variants.

## Graphical Abstract

**Graphical Abstract:**
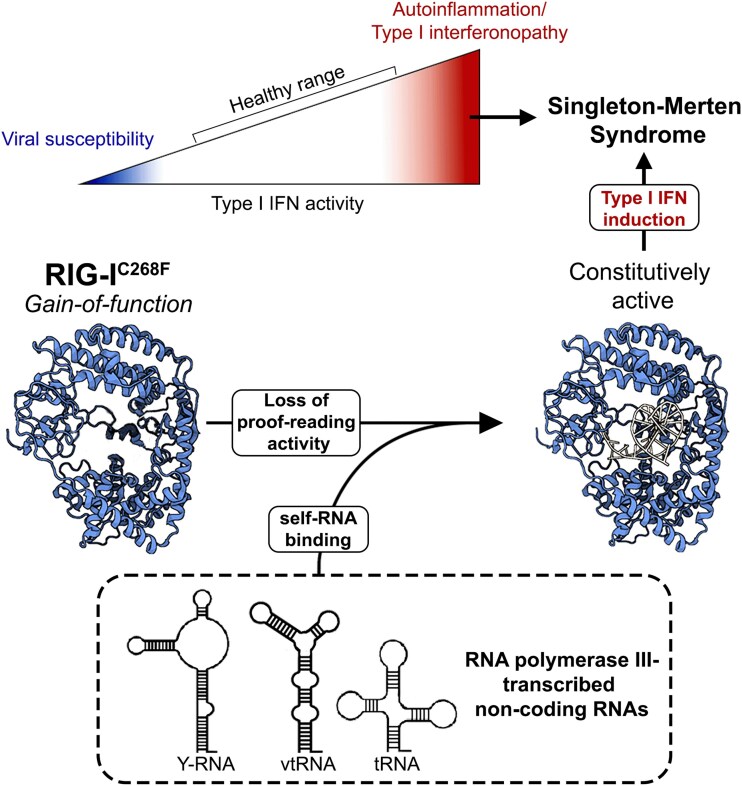

## Introduction

The innate immune system serves as an integral barrier between host and pathogens. It employs conserved receptors that detect microbial signatures and initiate rapid induction of inflammatory mediators and activation of immune cells. The type I interferons (IFNs) are key inflammatory cytokines that are produced and secreted upon activation of nucleic acid sensors. During viral infection, virus-derived nucleic acids are sensed by DNA and RNA receptors. In mammals, the major classes of RNA sensors are the endosomal Toll-like receptors, the RNA-sensing inflammasome, and the cytosolic RIG-I-like receptors (RLRs) [1].

There are three members of the RLR family. Retinoic acid-inducible gene I (RIG-I), melanoma differentiation-associated gene 5 (MDA5) and laboratory of physiology and genetics 2 (LGP2). These receptors share structural and domain similarities, and their activation via RNA ligand binding converges on the mitochondrial antiviral-signalling (MAVS) adaptor protein and subsequent type I IFN induction [1]. RIG-I preferentially binds highly structured, base-paired, shorter (<500 bp) RNA with a 5′ tri/diphosphate motif (5′ppp/pp) and preferably a blunt end [2]. These motifs are abundant on virus-derived RNAs and can also occur on self-derived (endogenous) RNAs transcribed by RNA polymerase III (RNAPIII), though such RNAPIII transcripts are subject to triphosphatase-mediated cleavage of the immunostimulatory 5′ppp [3]. MDA5 is known to bind to longer tracts of highly base-paired, double-stranded RNA (dsRNA) (>1000 bp), and is aided by LGP2 [4].

The RIG-I protein comprises two N-terminal caspase recruitment domains (2CARD), an internal DExD/H box helicase domain (Hel1, Hel2i, and Hel2) containing a proof-reading ATP-binding SF2 domain and a C-terminal regulatory domain (RD). In the absence of an RNA ligand, the 2CARDs are sequestered by the Hel2i domain preventing CARD oligomerisation and downstream signalling [5]. Upon binding of RNA to the RIG-I RD, the helicase domain changes conformation and forms an interface with the RNA molecule. This interaction is facilitated upon ATP binding and Mg^2+^-dependent hydrolysis by the SF2 domain and confers proof-reading activity to RIG-I, allowing it to verify the presence of a 5′ppp/pp motif [6]. This in turn releases the 2CARDs from their autoinhibitory state and permits 2CARD interaction with MAVS. ATP hydrolysis is a key mechanism through which RNA lacking a 5′ppp/pp motif is efficiently ejected from RIG-I. A weak interaction with either 5′ monophosphorylated (5′p) or 5′ hydroxyl (5′OH) RNAs and the RD leads to a distinctive conformational change in RIG-I triggering ATP hydrolysis and dissociation of the RNA by translocation [7, 8]. This ‘switch’ forms a critical regulatory brake in preventing aberrant activation of RIG-I by self-RNA ligands, downstream type I IFN induction, and the associated pathological consequences.

The type I interferonopathies are a growing collection of autoinflammatory inborn errors of immunity centred around the dysregulation of type I IFN production, sensing, or resolution [9]. Within this classification is Aicardi-Goutières syndrome, STING-associated vasculopathy in infancy, pseudo-TORCH syndrome, and Singleton-Merten syndrome (SMS). Though these disorders all have type I IFN dysregulation as the basis of their underlying pathophysiology, the manifestations of the diseases vary substantially [9]. SMS is an exceptionally rare and atypical type I interferonopathy resulting from gain-of-function (GoF) mutations within *RIG-I* or *IFIH1* (MDA5). Two SMS-causing variants impair RIG-I ATPase activity—RIG-I^C268F^ and RIG-I^E373A^ [10]. Patients with these GoF variants developed congenital glaucoma, osteoarthropathy, and aortic calcifications [11]. Both the RIG-I^C268F^ and RIG-I^E373A^ variants have been the focus of several studies due to their uniquely altered mechanisms, which have been instrumental in understanding the dynamics of RIG-I activation [7, 10, 12]. When expressed, these variants induce spontaneous constitutive type I IFN (IFN-β) expression without a viral infection or exogenous ligand [13]. The RIG-I^C268F^ variant results in a pseudo-‘ATP-bound’ state in which RIG-I is unable to bind and therefore hydrolyze ATP, resulting in stabilization and retention of self-RNA [10]. The RIG-I^E373A^ variant has reduced ATP hydrolysis despite functional ATP binding. An additional structurally-derived RIG-I^E373Q^ variant phenocopies RIG-I^E373A^ by locking RIG-I into its ATP-bound state via the same mechanism [13].

Despite extensive characterization of the molecular dynamics involved in RNA binding and activation, the understanding of the precise self-RNAs activating these SMS RIG-I variants remains incomplete. The functionally similar RIG-I^E373Q^ variant was co-purified with 28S ribosomal RNA (rRNA) [13]. The RIG-I^E373A^ variant was shown to interact with Y-RNAs, *RNY4* specifically, in kidney cells [12]. However, for the RIG-I^C268F^ variant there has been no definitive identification of immunostimulatory self-RNAs.

In response to viral infection, wild-type (WT) RIG-I binds a range of self-RNAs. During HIV-1 or measles virus infection, non-coding RNAs (ncRNAs) derived from RNAPIII, such as Y-RNAs, have been identified as endogenous ligands for RIG-I [14]. Similarly, during lytic reactivation of Kaposi’s sarcoma-associated herpesvirus and dengue virus infection, RIG-I binds RNAPIII-transcribed vault RNAs (vtRNAs) [14, 15]. These RNAPIII ligands share remarkable similarities to short virus-derived RNAs including their propensity for base pairing, complex secondary structures, and 5′ triphosphorylation. The importance of 5′ppp motifs has been demonstrated biochemically through phosphatase treatment of *in vitro* transcribed 5′ppp vtRNAs negating their immunostimulatory capacity [15]. During herpes simplex virus-1 (HSV-1) infection, expression and nucleocytoplasmic relocalization of endogenous RNAPIII-transcribed 5S ribosomal RNA pseudogene 141 (*RNA5SP141*) binds and activates RIG-I, allowing activation of RNA sensing pathways upon infection with a DNA virus [16]. The binding of *RNA5SP141* propagates anti-viral responses and its deletion hampers cellular immunity to HSV-1. Strikingly, patient mutations in the gene *GTF3A*, which encodes transcription factor IIIA that associates with the RNAPIII complex, results in decreased *RNA5SP141* expression that impairs innate immune responses to HSV-1 and predisposes to herpes simplex encephalitis [16].

While self-RNA recognition by RIG-I has been identified in the context of ongoing viral infection, the identity of self-RNAs bound in autoinflammatory disorders remains elusive—representing a crucial gap in understanding the pathologies of SMS and wider RIG-I-dependent autoinflammatory disorders. Characterizing the repertoire of self-RNAs that aberrantly engage RIG-I during sterile inflammation may explain why certain cell types and tissues exhibit selective vulnerability to sterile RNA sensing. In this study, we therefore used clinically relevant RIG-I GoF mutations (in particular RIG-I^C268F^) as a means to identify endogenous RNAs bound during spontaneous type I IFN signalling.

## Results

### SMS RIG-I gain-of-function variants induce a sterile type I IFN response

**Figure 1:**
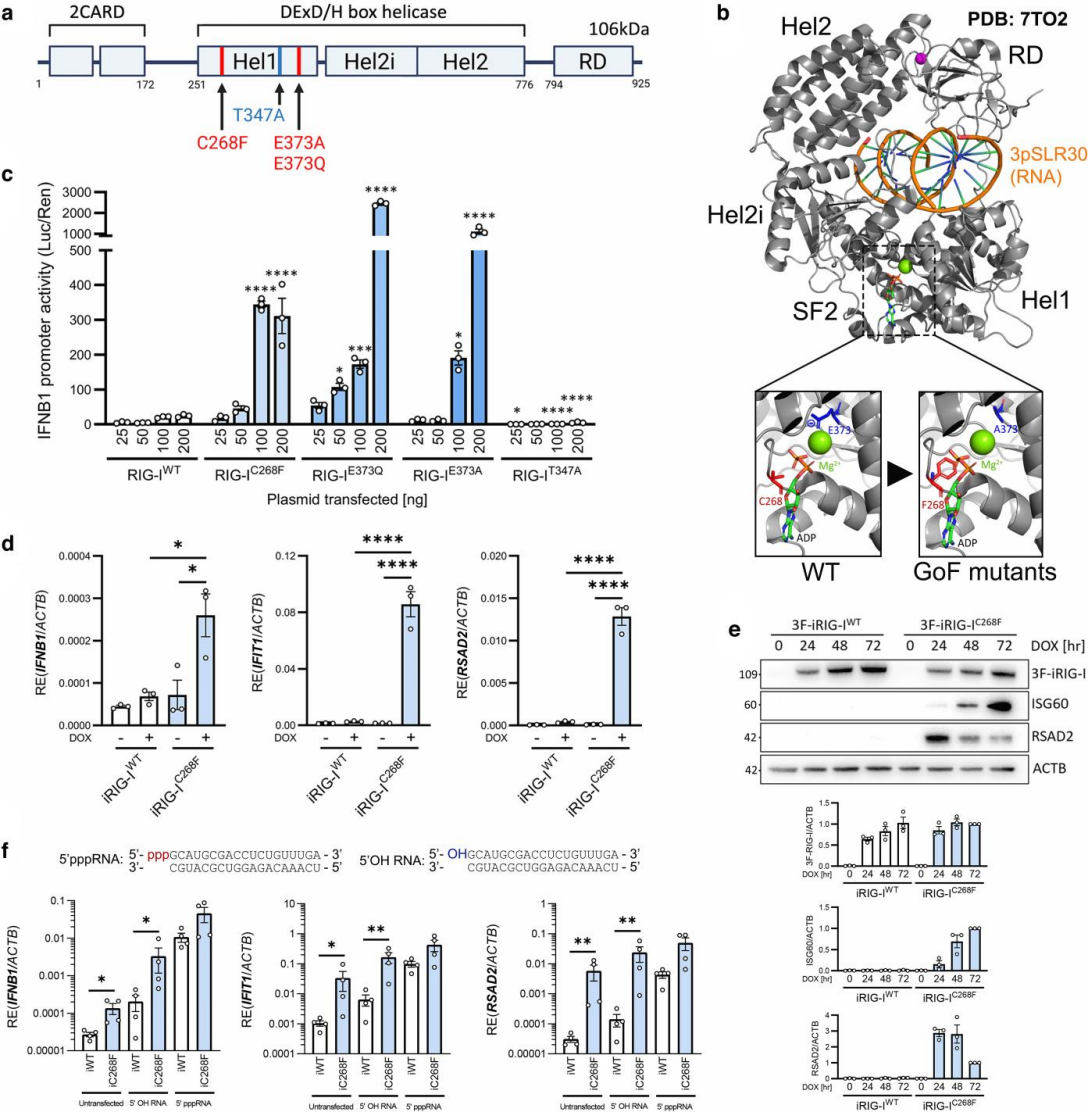
SMS RIG-I gain-of-function variants induce a sterile type I IFN response. (a) Schematic illustration of RIG-I protein highlighting domain architecture and gain-of-function (red-C268F/E373A/E373A) and RNA-binding mutations (blue-T347A). (b) Wild-type RIG-I cryo-EM structure resolved with 5′ triphosphorylated blunt-ended 30-bp RNA (3pSLR30), ADP, Mg^2+^, and Zn^2+^ (PDB: 7TO2). Expanded SF2 domain with positions of wild-type C268 and E373 residues, and SMS-mutant F268 and A373 residues, indicated. (c) *IFNB1*-luciferase reporter assay. HEK293 cells were co-transfected with indicated dose of pcDNA3.1-RIG-I vector, p125-Firefly Luciferase IFN-β reporter, and pRL-TK Renilla reporter for 24 hours. Firefly luciferase (Luc) activity was calculated by normalization to Renilla (Ren) activity. *n* = 3. A two-way ANOVA was performed with Šídák's multiple comparisons test. Data were compared with the RIG-I^WT^ control. (d) iRIG-I^WT^ and iRIG-I^C268F^ cells were treated with 1 μg/mL doxycycline for 48 hours followed by qPCR analysis for relative expression of *IFNB1*, *IFIT1*, and *RSAD2*. *n* = 3. An ordinary one-way ANOVA was performed. (e) iRIG-I^WT^ and iRIG-I^C268F^ cells were treated with 1 μg/mL doxycycline for 0, 24, 48 or 72 hours followed by western blot analysis for protein expression of 3FLAG-RIG-I, ISG60, and RSAD2. ACTB loading control. Densitometry analysis was performed and values per repeat were normalized to their respective 72 hr iRIG-I^C268F^ sample, set to 1. *n* = 3. (f) iRIG-I^WT^ and iRIG-I^C268F^ cells were treated with 1 μg/ml doxycycline for 24 hours followed by transfected with 500 ng of either 5′pppRNA or 5′OH RNA for 6 hours, followed by qPCR analysis for relative expression of *IFNB1*, *IFIT1*, and *RSAD2*. Data were log_10_ transformed and statistics was determined with ordinary one-way ANOVA. *n* = 4. All data are represented as means ± SEM. (* *P* < 0.05, ** *P* < 0.01, *** *P* < 0.001, **** *P* < 0.0001).

RIG-I^C268F^ and RIG-I^E373A^ (as well as the phenocopying RIG-I^E373Q^) are located within the Hel1 helicase domain (Fig. 1A). An additional previously characterized RNA-binding mutation in RIG-I (RIG-I^T347A^) is also found within the Hel1 domain at the structural interface between RIG-I and docked RNA (Fig. 1A) [13]. A resolved cryo-EM structure of human RIG-I (lacking the disordered 2CARD domain) in complex with 5′ppp base-paired RNA (p3SLR30) [PDB: 7TO2] highlights the spatial proximity of RIG-I^C268F^ and RIG-I^E373A^ to the ATP-binding site and the Mg^2+^ ion required for ATP hydrolysis. This proximity likely impacts the Mg^2+^-dependent hydrolytic activity necessary for the ejection of self-RNA (Fig. 1B).

To evaluate whether these GoF variants can induce spontaneous type I IFN expression, we transiently overexpressed WT and mutant RIG-I constructs in HEK293 cells and co-transfected an *IFNB1* promoter firefly luciferase reporter plasmid to monitor *IFNB1* induction (a direct read-out of type I IFN activity) and a constitutive *Renilla* luciferase expression plasmid for normalization purposes (Fig. 1C). Overexpression of RIG-I^WT^ led to a modest dose-dependent increase in *IFNB1* promoter activity due to autoactivation. In contrast, overexpression of RIG-I^C268F^, RIG-I^E373A^, and RIG-I^E373Q^ led to a robust dose-dependent activation of the *IFNB1* promoter. The RNA-binding mutant RIG-I^T347A^ variant failed to elicit *IFNB1* promoter activity. Among the GoF variants, RIG-I^C268F^ triggered the strongest *IFNB1* response at the lowest transfection dose (100 ng).

To confirm these findings under controlled expression conditions, we generated doxycycline-inducible RIG-I^WT^ or RIG-I^C268F^ (iRIG-I) lentiviral constructs, and transduced these into a RIG-I knockout (RIG-I^KO^) HEK293 line [17]. Following induction for 48 hours, iRIG-I^C268F^ showed a significant upregulation of *IFNB1* transcript, as well as elevated expression of two canonical interferon-stimulated genes (ISGs), namely *IFIT1* and *RSAD2* (Fig. 1D). This ISG induction was also observed at the protein level over a 72-hour time course. While both iRIG-I^WT^ and iRIG-I^C268F^ were expressed to similar levels, upregulation of ISG60 and RSAD2 proteins was observed only in iRIG-I^C268F^ cells (Fig. 1E).

The presence of a 5′ppp/pp motif on an RNA ligand is required for canonical RIG-I binding and activation [7]. As expected, transfection of 5′pppRNA into doxycycline-treated iRIG-I^WT^ cells induces a robust type I IFN response over a 6-hour time course, which is potentiated in doxycycline-treated iRIG-I^C268F^ cells (Fig. 1F). Transfection with 5′ppp dsRNA control (5′OH RNA) is not immunostimulatory as demonstrated by the lack of an IFN response after transfection into doxycycline-treated iRIG-I^WT^ cells. By contrast, transfection of 5′OH RNA into doxycycline-treated iRIG-I^C268F^ cells leads to a significantly upregulation of *IFNB1* transcript, reaffirming this variant’s inability to effectively differentiate between canonical and non-stimulating ligands.

Consistent with previous reports, we conclude that RIG-I autoinflammatory variants, most notably RIG-I^C268F^, show a spontaneous type I IFN response to self-RNA in the absence of a viral pathogen. In addition, the presence of a 5′ppp motif is not essential to activate RIG-I^C268F^, consistent with the loss of discriminatory activity.

### Both RIG-I^wt^ and RIG-I^c268f^ bind to RNA polymerase III-derived self-RNAs

**Figure 2:**
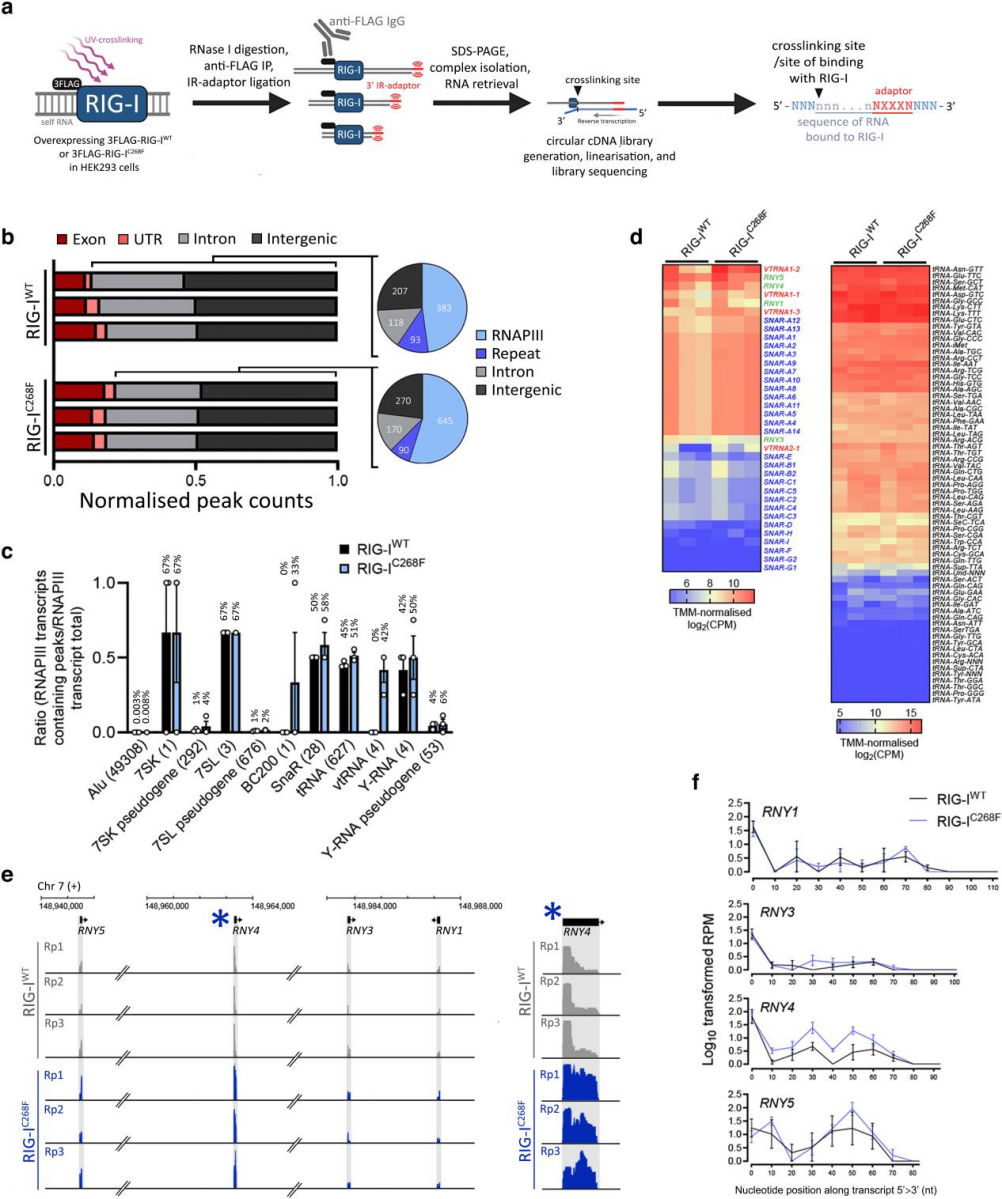
RIG-I^WT^ and RIG-I^C268F^ bind RNA polymerase III-derived self-RNAs. (a) Schematic of the irCLIP protocol used to identify RIG-I bound RNAs in HEK293 cells overexpressing 3FLAG-RIG-I^WT^ or 3FLAG-RIG-I^C268F^. UV-crosslinking and immunoprecipitation (IP and re-IP) were performed prior to irCLIP library assembly, sequencing, and peak calling. *n* = 3. Schematic designed on Biorender. (b) Distribution of irCLIP peaks across genomic regions (exon, intron, UTR, intergenic) and further classification of intron/intergenic peaks into RNAPIII-transcribed genes and repeat elements. (c) Percentage of RNAPIII-transcribed genes containing ≥1 peak, grouped by transcript class. Percentages averaged across triplicate are indicated above each bar. (d) Heatmaps showing CPM values (TMM-normalized in order to adjust irCLIP data for varied library sizes between replicates) for selected RNAPIII transcripts (*RNY*, *VTRNA*, *SNAR* and tRNA families) across triplicate samples of RIG-I^WT^ and RIG-I^C268F^. (e) JBrowse2 tracks of irCLIP read coverage across *RNY* locus in RIG-I^WT^ and RIG-I^C268F^ replicates. (f) Crosslinking site analysis showing irCLIP read density across *RNY* RNAs, highlighting preferential binding at the 5′ termini for both RIG-I variants.

To characterize the RNA species bound by RIG-I, we performed irCLIP on HEK293 cells transiently overexpressing either 3FLAG-RIG-I^WT^ or 3FLAG-RIG-I^C268F^ for 48 hours (Fig. 2A) [18, 19]. We chose to make use of a transient overexpression system to ensure the high RIG-I expression for efficient immunoprecipitation (IP). UV irradiation covalently linked RIG-I to associated RNAs before lysis, preserving native RNA-protein interactions during the subsequent isolation. Lysates were subjected to partial RNase I digestion to obtain optimal fragment length (50–500 bp), followed by anti-FLAG IP and stringent purification. After RIG-I retrieval, the bound RNA was conjugated to an infrared 3′ adaptor sequence that allowed for visualization after electrophoresis and sample barcoding. To reduce background prior to SDS-PAGE, a secondary FLAG IP (re-IP) was performed on denatured RIG-I:RNA complexes. Next, RIG-I:RNA complexes were separated by gel electrophoresis and smears above the molecular weight of 3FLAG-RIG-I (110 kDa) were extracted (Supplementary Fig. 1A). Extracted RNA was transcribed into a cDNA library, and DNA species (between 150 and 500 bp) were excised for downstream high-throughput sequencing (Supplementary Fig. 1B). The reads generated from the sequencing of these irCLIP libraries provide nucleotide-level resolution, with the end of reads clustering around RIG-I binding sites. *PureCLIP* was used to call the statistical likelihood that a cluster of reads could be denoted as a positive RIG-I binding site or peak [20].

RIG-I binding sites detected as irCLIP peaks were spread across multiple genomic regions, including exons, introns, untranslated regions (UTRs), and intergenic loci. Both RIG-I^WT^ and RIG-I^C268F^ exhibited similar distributions across these categories, with minimal variation between replicates (Fig. 2B). Further analysis of intronic and intergenic peaks using a recently developed database annotating RNA polymerase III-transcribed ncRNAs (Pol3Base) revealed that approximately half of these peaks corresponded to RNAPIII-transcribed genes, while a smaller fraction aligned to repeat elements (Fig. 2B) [21].

The RNAPIII-associated peaks were further classified by class. We calculated the proportion of genes within each RNAPIII class that contained at least one irCLIP peak. Peaks were observed across the majority of RNAPIII-transcribed genes, with comparable peak calling between RIG-I^WT^ and RIG-I^C268F^ (Fig. 2C). Approximately 50% of the 627 annotated tRNA genes contained RIG-I binding peaks, however RNAPIII-transcribed independent *Alu* elements (excluding RNAPII-encoded *Alu* elements contained within coding regions or UTRs) showed very low peak frequency relative to their genomic abundance (Fig. 2C).

Across the RNAPIII transcript classes some individual genes were more enriched on RIG-I^WT^/RIG-I^C268F^ than others (Fig. 2D). Within the family of small NF90 (nuclear factor 90)-associated RNA (SnaR), *SNAR-A* read counts were consistently high in both RIG-I^WT^ and RIG-I^C268F^ datasets, in comparison to other SnaR members that showed lower read counts. Y-RNAs and vtRNAs (with the exception of *VTRNA2-1*) displayed uniformly high trimmed mean of M (TMM) values across all members. In contrast, read depth was highly variable across the tRNA family.

Genome browser visualisation of irCLIP aligned reads across *RNY* (Y-RNA) (Fig. 2E), *VTRNA* genes, and two tRNA^Pro^ loci (Supplementary Fig. 1C/1D) confirmed that irCLIP reads were confined to annotated gene bodies, with minimal signal in flanking regions. Notably, *RNY4* exhibited a pronounced 5′ binding preference (Fig. 2E). irCLIP analysis allows precise mapping of binding sites at single-nucleotide resolution [19]. Aggregated crosslinking site analysis across *RNY1–5* revealed consistent and robust enrichment at the 5′ termini, with a smaller frequency of peaks detected in central (stem) regions (Fig. 2F). Crosslinking site analysis was also performed for the *VTRNA* genes emphasizing more distributed RIG-I binding sites (Supplementary Fig. 1E), and across tRNA families highlighting both 5′ terminus and centralized binding (exemplified for the tRNA^His^ family in Supplementary Fig. 1F).

Contrary to our expectation that RIG-I^C268F^ would uniquely bind either a greater abundance of RNAs or unique RNAs, the irCLIP showed that both RIG-I^WT^ and RIG-I^C268F^ had very similar binding patterns to their associated RNAs. In conclusion our irCLIP study revealed that both RIG-I^WT^ and RIG-I^C268F^ associate with RNAPIII transcripts, most notably, Y-RNAs.

### Y-RNAs effectively and uniquely associate with RIG-I^c268f^

**Figure 3:**
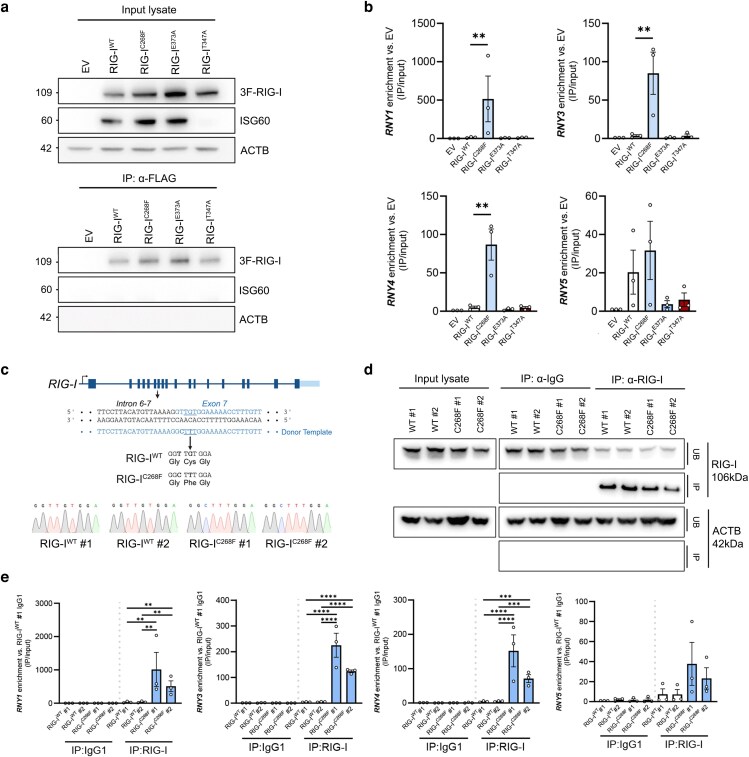
Y-RNAs effectively and uniquely associate with RIG-I^C268F^. (a) Western blot after FLAG-IP performed on lysates from HEK293 cells overexpressing 3F-RIG-I (WT/C268F/E373A/T347A) for 48 hours. *n* = 3. (b) Native RIP-qPCR analysis of RNY RNAs bound to RIG-I. Data is expressed as a fraction of input *RNY* levels. Enrichment of RNYs was then calculated by normalization to the empty vector control. Statistical significance was assessed by one-way ANOVA on log_10_-transformed data. *n* = 3. (c) Strategy of CRISPR-editing RIG-I^C268F^ variant into *MAVS*^KO^ A549 cells (including PAM-site annulling mutation to prevent repeated editing). Sequencing chromatograms from two RIG-I^WT^ and two RIG-I^C268F^ selected clones. (d) Western blot confirmation after RIG-I-IP performed on lysates from A549 cells expressing endogenous RIG-I (WT/C268F clones) after 24 hours stimulation with 500 U/mL IFNa/d. *n* = 3. (e) Endogenous RIP-qPCR analysis of RNY RNAs bound to RIG-I. Data are expressed as a fraction of input *RNY* levels. Enrichment of RNYs was then calculated by normalization to the RIG-I^WT^ #1 IgG control. Statistical significance was assessed by one-way ANOVA on log_10_-transformed RIG-I IP data. *n* = 3. All data are represented as means ± SEM. (* *P* < 0.05, ** *P* < 0.01, *** *P* < 0.001, **** *P* < 0.0001).

To validate the findings from the irCLIP, we performed native RNA immunoprecipitations (RIP). Native RIPs do not use crosslinking and rely on intermolecular forces to keep the target protein and RNA in proximity during the protocol. HEK293 cells were transiently transfected with 3FLAG-RIG-I (WT, C268F, E373A, or the RNA-binding mutant T347A) for 48 hours before lysis. SDS-PAGE analysis was used to verify pulldown of 3FLAG-RIG-I and showed spontaneous ISG60 expression in samples overexpressing RIG-I^WT^/RIG-I^C268F^/RIG-I^E373A^. The ISG60 response in 3FLAG-RIG-I^WT^ most likely due to the high level of RIG-I expressed and CARD-mediated autoactivation (Fig. 3A). Analysis of bound RNAs by qPCR found significant enrichment of Y-RNAs (with the exception of *RNY5*) bound to 3FLAG-RIG-I^C268F^ compared to 3FLAG-RIG-I^WT^ (Fig. 3B). These observations differed from the irCLIP that suggested comparable Y-RNA interactions between RIG-I^WT^ and RIG-I^C268F^. Additionally, of the vtRNA genes only *VTRNA1-1* was significantly enriched on 3FLAG-RIG-I^C268F^ compared to 3FLAG-RIG-I^WT^ (Supplementary Fig. 2A). Interestingly, in our native-RIP, the 3FLAG-RIG-I^E373A^ variant did not seem to enrich for any of the selected target RNAs despite the variant inducing an IFN response.

As mentioned, the RIG-I^C268F^ variant mechanistically locks RIG-I in a pseudo-‘ATP-bound’ state preventing the ejection of undesirable RNA ligands. We hypothesized that the RIG-I^C268F^ variant acted in an analogous manner to the crosslinking step of the irCLIP, allowing for RNA-binding but inhibiting self-RNA dissociation. This results in the retention of *RNY* RNAs to RIG-I^C268F^ even in the absence of RNA-protein crosslinking. Instead, at steady-state these RNAs dissociate from RIG-I^WT^ in the absence of a crosslinking reaction. In line with this hypothesis, introduction of a formaldehyde-crosslinking step in the RIP protocol revealed that both RIG-I^WT^ and RIG-I^C268F^ are able to associate with *RNY* RNAs (Supplementary Fig. 2B/2C). In the case of RIG-I^WT^, this interaction is likely to be transient, and RIG-I will dissociate from the RNA during ATPase-dependent proofreading [22].

Both the irCLIP and subsequent RIP relied on the overexpression of 3FLAG-RIG-I. In order to validate that the overexpression of RIG-I was not producing artefactual binding of RIG-I to RNAPIII transcripts we performed an endogenous RIG-I RIP. Acquisition of patient cells expressing RIG-I^C268F^ was not possible, therefore CRISPR/Cas9-editing was performed to introduce the RIG-I^C268F^ mutation into endogenous RIG-I locus in A549 cells (Fig. 3C). We used MAVS-deficient (MAVS^KO^) A549 cells (generated through CRISPR/Cas9-editing; Supplementary Fig. 2D) in order to prevent potentially deleterious (and cell-growth limiting) spontaneous type I IFN signalling upon editing of *RIG-I* locus to introduce the RIG-I^C268F^ mutation. We validated that the selected MAVS^KO^ cell line was unresponsive to transfection with polyI:C (Supplementary Fig. 2E). Correct editing of the *RIG-I* locus was confirmed by Sanger sequencing of the edited region (Fig. 3C). Two RIG-I^WT^ and two RIG-I^C268F^ single-cell clones were selected for similar growth rate and comparable expression of RIG-I and responsiveness to recombinant type I IFN (Supplementary Fig. 2F). These cells were pretreated with recombinant type I IFN to increase expression of RIG-I and to improve IP efficiency. Efficient pulldown of endogenous RIG-I with a polyclonal antibody was demonstrated by SDS-PAGE and immunoblotting (Fig. 3D). Endogenous RIG-I RIP identified significant enrichment of *RNY1*, *RNY3*, and *RNY4* with both RIG-I^C268F^ clones when compared to RIG-I^WT^. Similar to the findings from the native RNA IP, *RNY5* was not significantly enriched (though an increased trend was observed) (Fig. 3E). Furthermore, there was no significant enrichment of either *VTRNA1-1* or *VTRNA1-2* with RIG-I in either RIG-I^C268F^ clone (Supplementary Fig. 2G). SnaR-A showed a small enrichment, while *tRNA-Pro-TGG* was significantly enriched on RIG-I^C268F^ compared to RIG-I^WT^ (Supplementary Fig. 2G–H).

When combined, we conclude from these experiments that *RNY1*, *RNY3*, and *RNY4* are consistently enriched upon RIG-I in RIP experiments, replicating the findings from the irCLIP. Although we see other RNA species binding RIG-I, these interactions are less consistently observed. For example, although vtRNAs were detected in the irCLIP and FLAG-RIG-I^C268F^ RIP, no vtRNAs were found to interact with endogenous RIG-I^C268F^. This underscores the importance of further validating the protein-RNA interactions identified through irCLIP, at endogenous protein level.

### Reducing *RNY* expression does not lessen spontaneous RIG-I^c268f^ signalling

Our irCLIP and subsequent RIPs indicated a role for RNAPIII-transcribed RNAs in specifically binding and activating RIG-I^C268F^. Though these techniques have highlighted the breadth of self-RNAs bound to RIG-I, a limitation of this approach is the inability to decipher whether single RNAs, families of RNAs, or a larger excess of RNAPIII-transcribed RNAs engage in driving the spontaneous activation of RIG-I^C268F^. There are technical difficulties associated with depleting ncRNAs due to their high abundance, high sequence homology, and crucial functionality within cellular pathways (such as with tRNAs). Recent literature has described the binding of Y-RNAs to both RIG-I^WT^ during viral infection and to the SMS-variant RIG-I^E373A^ [12, 14]. However, the role of Y-RNAs in mediating RIG-I^C268F^ driven inflammatory responses has not yet been elucidated. Since we observed strong binding between RIG-I^C268F^ and *RNY1*/*3*/*4* in both the irCLIP and RIPs, we decided to further investigate whether Y-RNAs are potential drivers of RIG-I^C268F^ activation and signalling.

**Figure 4:**
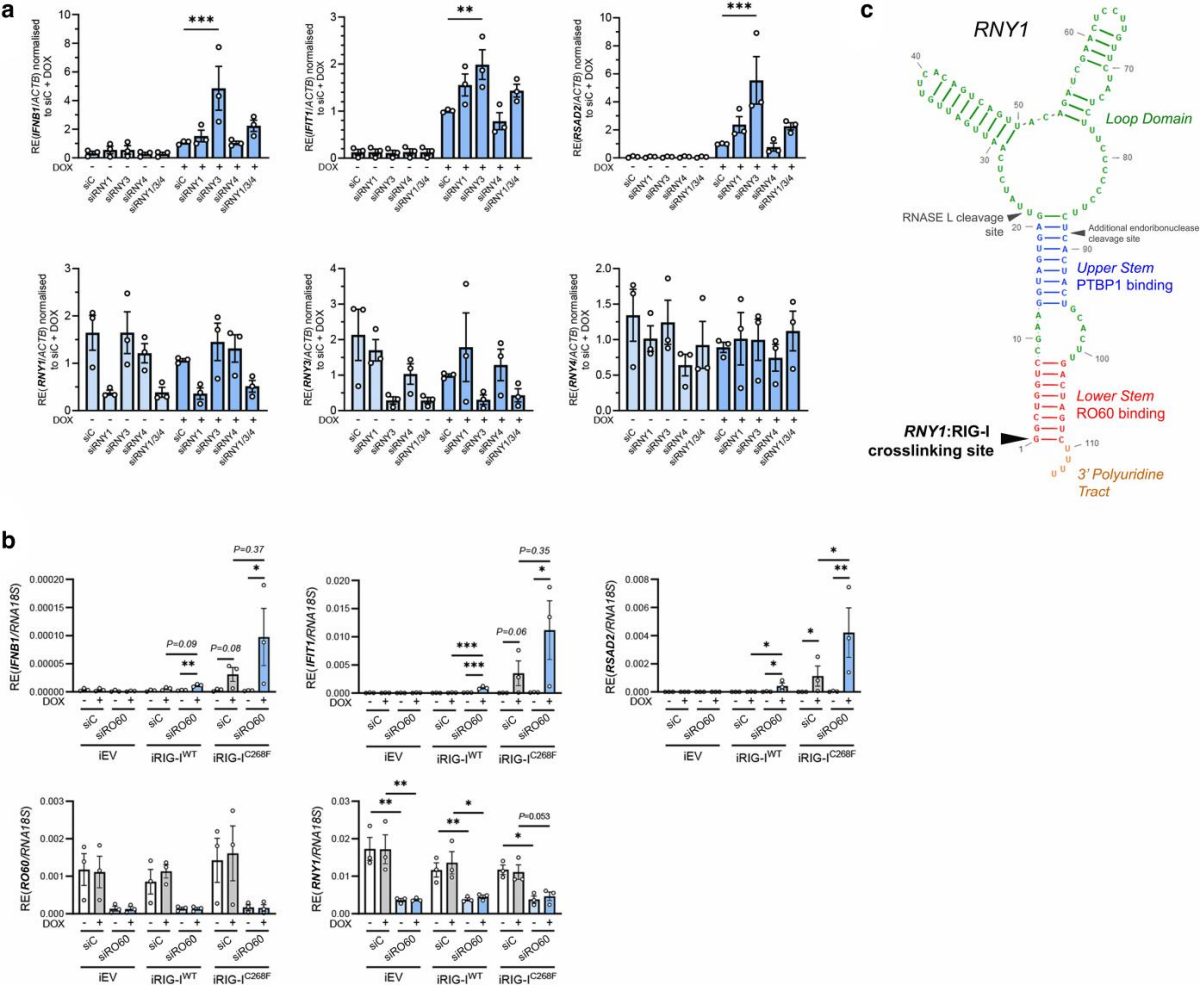
Manipulation of RNYs does not impact spontaneous RIG-I^C268F^ signalling. (a) Knockdown of either *RNY1*, *RNY3*, *RNY4*, or in combination (*RNY1*/*3*/*4*) in HEK293 iRIG-I^C268F^ cells. Cells were transfected with respective siRNA for 72 hours, and for the final 24 hours of knockdown cells were treated with 1 μg/ml doxycycline to induce RIG-I^C268F^ expression. RNA was isolated for qPCR analysis of *IFNB1*, *IFIT1*, and *RSAD2* expression. *n* = 3. Statistical significance was assessed by one-way ANOVA. Data normalized for each biological replicate to cells treated with siC and with doxycycline. *n* = 3. (b) Knockdown of *RO60* in HEK293 iEV, iRIG-I^WT^, or iRIG-I^C268F^ cells. Cells were transfected with respective siRNA for 72 hours, and for the final 24 hours of knockdown cells were treated with 1 μg/mL doxycycline to induce RIG-I expression. RNA was isolated for qPCR analysis of *IFNB1*, *IFIT1*, and *RSAD2* expression. *n* = 3. Statistical significance was assessed by one-way ANOVA. (c) Predicted secondary structure of *RNY1* highlighting regions of interest including RIG-I crosslinking site as determined by irCLIP and known *RNY1* cleavage sites. All data are represented as means ± SEM. (*P < 0.05, **P < 0.01, ***P < 0.001, ****P < 0.0001).

Small interfering RNAs (siRNAs) targeting *RNY1*/*RNY3*/*RNY4* individually or in combination as a pool (siRNY1/3/4) were transfected into iRIG-I^C268F^ HEK293 cells for 48 hours, followed by 24 hours of doxycycline-mediated RIG-I^C268F^ induction (Fig. 4A). *RNY* knockdown efficiency and induction of IFN and ISGs was determined by qPCR. Unexpectedly, depletion of *RNY3* in the presence of RIG-I^C268F^ modestly, but significantly, increased expression of *IFNB1*, *IFIT1,* and *RSAD2*. Depletion of *RNY1*, *RNY4*, or the combination of *RNY1*/*RNY3*/*RNY4* did not significantly impact type I IFN induction by RIG-I^C268F^. These findings are limited by the inadequate knockdown efficiency of *RNY4*.

To circumvent the incomplete siRNA-mediated knockdown of Y-RNAs, we explored an alternative approach to deplete Y-RNA expression. RO60 is a ribonucleoprotein (RNP) that binds and stabilizes Y-RNAs within both the nucleus and cytoplasm [23]. Nuclear association of Y-RNAs with RO60 facilitates their export and supports diverse functions, including subcellular localization and extracellular vesicle packaging [24]. The stabilizing role of RO60 is well established, and its deletion has been shown to trigger exosome-mediated degradation of free cytoplasmic Y-RNAs [25, 26]. Leveraging this property, we sought to manipulate Y-RNA expression levels in iRIG-I HEK293 cells. As expected, siRNA-mediated knockdown of RO60 resulted in a significant reduction in detectable *RNY* transcripts, confirming its critical role in maintaining Y-RNA stability (Fig. 4B). Surprisingly, depletion of RO60 lead to an increase in *IFIT1* and *RSAD2* expression after doxycycline-mediated induction of RIG-I^WT^ and iRIG-I^C268F^. As RO60 depletion leads to a reduction in *RNY* levels, the stimulatory consequences to type I IFN signalling were contrary to expectation.

Taken together, these findings indicate that despite Y-RNAs readily associating with RIG-I^C268F^, their partial depletion does not diminish spontaneous RIG-I^C268F^ signalling and may even unmask alternative self-RNA interactions that enhance pathway activation. Alternatively, the loss of RO60 may expose a binding site on Y-RNAs that allows increased RIG-I binding (Fig. 4C), despite lower Y-RNA abundance. Additionally, these data imply that other endogenous RNAs are likely partaking in the constitutive activation of RIG-I^C268F^.

## Discussion

SMS is a rare autoinflammatory disease characterized by unrestrained, sterile type I IFN signalling driven by GoF variants within RIG-I and MDA5 [11]. These variants exemplify the consequences of an inability to distinguish self- from non-self RNA. In the absence of viral ligands, cells carrying RIG-I variants such as RIG-I^C268F^ spontaneously signal in an RNA-dependent manner, as the receptor is locked in a pseudo-‘ATP-bound’ state and unable to release non-productive RNA ligands [10, 27]. While the structural mechanics of RIG-I^C268F^ have been well defined, the identity of the endogenous immunostimulatory RNAs responsible for its activation has remained elusive.

Using irCLIP and RIP approaches, our study revealed a broad repertoire of RNAPIII-derived ncRNAs bound to RIG-I^C268F^. These included tRNAs, vtRNAs, and Y-RNAs. Notably, these RNA families share structural features with viral RNA ligands, such as the propensity to fold into complex secondary structures with extended base-paired nucleotide tracts. Unlike conventional m^7^G-capped transcripts, RNAPIII-derived RNAs are synthesized with a 5′ triphosphate motif, which can be processed by RNA phosphatases such as DUSP11 [3]. This biochemical property may further influence their recognition and immunostimulatory potential.

Along with our data regarding RIG-I^C268F^, multiple studies have identified RNAPIII transcripts, in particular Y-RNAs, as RIG-I interactors [12, 14, 28]. Y-RNAs are expressed ubiquitously, though to varying degrees across tissues, and are known to have strongest expression in the brain, heart and muscle [29]. The biological function of Y-RNAs appears diverse, ranging from assembly of RNP complexes to cell-to-cell communication [23, 30]. Y-RNAs are exported from the nucleus to the cytoplasm via their interaction with RO60, and RO60/RNY complexes have been associated with type I IFN-driven autoinflammatory diseases such as SLE [31, 32]. Our irCLIP and RIP experiments found the strongest interactors between the Y-RNA family and RIG-I^C268F^ were formed by *RNY1*, *RNY3*, or *RNY4*. Though enriched, *RNY5* was not significantly bound to RIG-I^C268F^ exclusively. Interestingly, the terminal base-pair of the double-stranded stem domain in *RNY1*/*RNY3*/*RNY4* is a G-C/U duplex as opposite to an A-U duplex in *RNY5*. RIG-I^WT^ has an approximately 2-fold increased affinity for terminal G-C pairs compared to terminal A-U pairs [33]. Additionally, the 3′ polyuridine overhang of *RNY5* is markedly longer than the other Y-RNAs. A longer 3′ overhang reduces RIG-I affinity multiple fold, which may explain the weaker interaction between *RNY5* and RIG-I^C268F^ [33]. The lower stem domain of *RNY1*/*RNY3*/*RNY4* where RIG-I is bound overlaps with the RO60 binding site [25]. We observe that loss of RO60 leads to an increase in type I IFN induction, despite decreased Y-RNA abundance. A reduction in Y-RNAs may allow more immunostimulatory self-RNAs to engage RIG-I in the absence of Y-RNA binding competition. Alternatively, the loss of RO60 may lead to increased RIG-I binding availability within the pool of remaining, yet diminished, Y-RNAs.

Transgenic mice expressing the human patient variant RIG-I^E373A^ spontaneously develop psoriasis-like skin lesions and nephritis at 15 weeks of age, with an incidence of 30% and 100% respectively [12, 34]. The kidneys of these mice exhibit interstitial inflammation, marked by increased expression of inflammatory cytokines and ISGs, immune cell infiltration, and renal dysfunction. RIP targeting ectopically expressed RIG-I^E373A^ in HEK293 cells, followed by sequencing, revealed enrichment of Y-RNAs (in particular *RNY4*) and many SnaR family members on RIG-I^E373A^ [12]. The enrichment of Y-RNAs is consistent with our findings for RIG-I^C268F^ (yet not for RIG-I^E373A^, perhaps due to minor experimental differences). While siRNA-mediated depletion of Y-RNAs did not diminish IFN induction in our experiments with RIG-I^C268F^ (possibly due to incomplete knockdown efficiency), the authors of this study demonstrated that CRISPR/Cas9-mediated knockout of *RNY4* abrogates spontaneous activation of RIG-I^E373A^. This observation may be due to RIG-I^C268F^ binding a broad repertoire of RNAPIII-transcribed RNAs, hence the depletion of Y-RNAs alone is not sufficient to reduce type I IFN responses.

During lytic reactivation of KHSV, endogenous vtRNAs are enriched on RIG-I^WT^, restricting viral activation [15]. *VTRNA1-1*, *1-2*, and *1-3* were identified to interact with RIG-I^C268F^ in our irCLIP and in ensuing RIP-qPCR experiments, however whether they have a role in promoting RIG-I^C268F^ activation remains unexplored. Not all RNAPIII-transcribed RNAs were identified as binding to RIG-I^C268F^, suggesting a degree of binding specificity. *Alu* elements, which can form long duplexed hairpin structures, are well established as potent activators of MDA5 gain-of-function variants [35]. In the context of TDP-43 deficiency, *Alu*-derived double-stranded RNAs accumulate and act as immunostimulatory ligands in a RIG-I-dependent manner [28]. However, our irCLIP analysis revealed only a very low proportion of RNAPIII-transcribed *Alu* elements were bound to RIG-I, indicating that these sequences may not represent primary ligands in this context. Similarly, while HSV-1 infection promotes binding of *RNA5SP141* to RIG-I^WT^ [16], we did not detect *RNA5SP141* associated with either RIG-I^WT^ or RIG-I^C268F^ in our irCLIP dataset, potentially reflecting the absence of HSV-1 infection in our experimental model. Finally, although SnaR RNA family members were identified in our irCLIP analysis, they did not appear to be strong RIG-I^C268F^ interactors in further downstream validation RIPs, despite a recent report describing SnaR RNAs (alongside Y-RNAs) as RIG-I^E373A^ interactors [12].

The expression profile and free availability of RNAPIII-derived transcripts vary considerably between cell lines and across different tissues [36, 37]. Such heterogeneity may represent a principal factor in the aetiology and pathophysiology of RLR-driven disorders, including SMS, which exhibits an atypical clinical presentation compared to other type I interferonopathies. Though Y-RNAs are broadly expressed across tissues, subtle differences in their abundance and distribution may significantly influence their binding to RIG-I^C268F^ [38]. For example, cardiac defects are a prominent feature of SMS and Y-RNA expression is known to be notably higher in heart tissue [39]. These observations underscore the importance of investigating self-RNA/RLR interactions in diverse cellular contexts, as such studies may reveal how self-RNA ligand availability and expression contributes to receptor-specific immune activation.

A significant advantage of using irCLIP over RIPs is the spatial information regarding the site of interaction along the target RNA. Our data show that RIG-I binding to RNAPIII-derived RNAs predominantly occurs at the 5′ termini of the RNA. This concurs with the understanding that RIG-I preferentially binds to the terminus of RNA molecules prior to either activation or ejection [22]. However, we have also observed RIG-I binding within internal regions of RNAPIII-transcripts, for instance within *RNY4* and *tRNA*^His^ genes. It is known that some ncRNA species are subject to endoribonuclease-mediated cleavage, for example by RNase L, leading to small duplexed RNAs [40]. This has been demonstrated for Y-RNAs (Fig. 4C) and tRNAs [41, 42]. In the case of RNase L-mediated cleavage, polyI:C stimulation led to the generation of products bearing a 2′,3′-cyclic phosphate motif. These 2′,3′-cyclic phosphates are known to act like 5′ppp motifs in promoting RIG-I binding [43]. Curiously, a number of internal RIG-I crosslinking sites that we identified align perfectly with the ‘new’ 5′ termini that are created upon RNAse L cleavage. The relationship between RIG-I GoF variants and RNAse L cleavage products is an interesting area for further investigation.

The ascribed variants associated with type I interferonopathies such as RIG-I^C268F^ in SMS delineate the upper limits of hyperactive type I IFN signalling in disease. These syndromes are complex, progressive, and associated with high mortality. Dysregulated type I IFN activity is a hallmark across a broader spectrum autoinflammatory and neuroinflammatory disorders, including rheumatoid arthritis, systemic lupus erythematosus, and neuromyelitis optica [44–46]. Genome-wide association studies have further implicated polymorphisms within the RLR genes that increase susceptibility to type I IFN-driven autoinflammatory diseases [47–49]. Given that RIG-I^C268F^ enhances interactions with self-RNA, it is plausible that additional, less-deleterious, variants within RIG-I may similarly predispose to aberrant signalling upon recognition of self-RNA, thereby driving low-level tonic type I IFN activity and contributing to the pathogenesis of more common autoinflammatory disorders. Advancing our understanding of the self-RNA ligands that activate gain-of-function RIG-I may provide critical insight into the mechanisms driving a much more diverse range of type I IFN-associated disorders.

## Materials and methods

### Cell culture and reagents

HEK293, HEK293T, and A549 (gift from Rob Hoeben, LUMC, Leiden) were cultured in Dulbecco’s modified Eagle’s medium (DMEM; Gibco, Thermo Fisher Scientific), supplemented with 10% heat-inactivated foetal bovine serum (Capricorn), 2 mM L-glutamine and 100 U/ml penicillin/streptomycin (Gibco, Thermo Fisher Scientific). Universal Type I IFN (IFN A/D; 11200, PBL Assay Science) was used at 500 U/ml unless stated otherwise. 5′ppp dsRNA, 5′ppp dsRNA control, and polyI:C LMW (Invivogen) were transfected with Lipofectamine 2000 (Invitrogen) at a 1:3 ratio according to the manufacturer’s protocol and was used at the indicated dosages. For antibiotic selection, puromycin (Sigma), geneticin (Invivogen), and hygromycin B (Roche) were used at concentrations mentioned below. Doxycycline hexahydrate (Sigma) was used at 1 μg/ml.

### Plasmids, siRNA, and transfection

Generation of pcDNA3.1 plasmids encoding 3FLAG-RIG-I variants (C268F, E373Q, E373A, T347A) were as follows. Site-directed mutagenesis (SDM) of full length human 3FLAG-RIG-I pcDNA3.1 (kindly gifted by Caetano Reis e Sousa/Jan Rehwinkel) using QuickChange II XL (Agilent Technologies) according to manufacturer’s protocol. Primers for SDM are listed (Supplementary Table. 6). All plasmids were transfected using Lipofectamine 2000 (Invitrogen) at a 1:3 ratio according to manufacturer’s protocol. For silencing of protein-coding genes, SMARTpool siGenome siRNAs against human RO60 (M-017733-02-0010), and a non-targeting control (D-001210-04-20) were used (Horizon Discovery). Cells were transfected using DharmaFECT 1 reagent (Horizon Discovery) at a final concentration of 25 nM according to manufacturer’s protocol.

### Generation of MAVS knockout using CRISPR-Cas9

Single Alt-R crRNA targeting MAVS was designed using the IDT design software (Supplementary Table. 3) (IDT, Integrated DNA Technologies). All subsequent Alt-R reagents were purchased from IDT. 5 μl of 200 μM Alt-R crRNA and 5 μl of 200 μM Alt-R tracrRNA were duplexed at 95°C for 5 minutes. RNP complexes were formed by mixing 1.2 μl of crRNA:tracrRNA duplex (final concentration 120 pmol), 2.2 μl Alt-R *S.p*. Cas9-GFP Nuclease (final concentration 104 pmol), and 2.1 μl PBS with incubation at room temperature for 20 minutes. For electroporation, 2 × 10^5^ A549 cells were resuspended in 16.4 μl SF solution supplemented with 3.6 μl of Supplement-1 (Lonza Bioscience). 5 μl of RNP complex and 1 μl of 100 μM Alt-R Electroporation Enhancer was added to A549 suspension. A549 cells were subject to electroporation with 4-D Nucleofector Unit (Lonza Bioscience) using the DN-100 programme and were immediately added to pre-warmed DMEM. The following day, GFP positive cells were single-cell sorted into 96-well format for clonal expansion and genotyping.

### Generation of RIG-I^c268f^ point mutation using CRISPR-Cas9

Single Alt-R crRNA targeting RIG-I around c.801T and homology directed repair (HDR) template were designed using the IDT design software (Supplementary Table. 3). Formation of RNP complex and electroporation were performed the same as the generation of the MAVS knockout line above, with the following additional steps. For electroporated cells, DMEM was prewarmed with the addition of 1.5 μl Alt-R HDR Enhancer per 1 ml DMEM. For electroporation, 2 × 10^5^ A549 cells were resuspended in 16.4 μl SF solution supplemented with 3.6 μl of Supplement-1 (Lonza Bioscience). 5 μl of RNP complex, 1.2 μl of 100 μM Alt-R Electroporation Enhancer, 1.2 μl of 100 μM Alt-R HDR template, and 2.6 μl PBS were added to A549 suspension. A549 cells were subject to electroporation with 4-D Nucleofector Unit using the DN-100 programme and were immediately added to pre-warmed DMEM. The following day, GFP positive cells were single-cell sorted into 96-well format for clonal expansion and genotyping.

### Genomic PCR

For confirmation of editing, genomic DNA was isolated from 4 × 10^4^ cells with 80 μl Extracta extraction buffer (Quantabio) and incubated at 95°C for 30 minutes. PCR was performed using 2X Phusion Plus PCR Master Mix (Thermo Fisher Scientific) with primers listed (Supplementary Table. 4). Sanger sequencing performed by the Leiden Genome Technology Center and visualized with SnapGene software.

### Lentivirus production and transduction

Full-length human RIG-I^WT^ and RIG-I^C268F^ were inserted into doxycycline inducible pLVX-Tight-Puro vector with N-terminal 3FLAG using NEBuilder (New England Biolabs) (pLVX-Tight-Puro_3F-RIG-I). For lentivirus production, HEK293T cells were transfected with pLVX-Tight-Puro_3F-RIG-I, psPAX2, and pMD2.G and lentivirus was collected after 48 hours. Stably integrated HEK293 cells (iEV/iRIG-I^WT^/iRIG-I^C268F^) were generated by transduction with lentivirus and selection with 1 μg/ml puromycin.

### RNA isolation, first-strand cDNA synthesis, and qPCR

Total cellular RNA was extracted using TRIzol/TRIzol LS (Invitrogen) according to manufacturer’s protocol. 500–1000 ng RNA was incubated with ezDNase (Invitrogen) for 10 minutes followed by cDNA synthesis with SuperScript IV VILO Master Mix (Invitrogen) according to the manufacturer’s protocol. cDNA was diluted in nuclease-free water and gene expression was measured using PowerUp SYBR Green Master Mix in technical duplicates on a QuantStudio 3 (Thermo Fisher Scientific). Gene-specific qPCR primers are listed (Supplementary Table. 2).

### RNA immunoprecipitation

HEK293 or A549 cells were seeded in 10 or 15 cm culture dishes respectively in which one culture dish was used per RIP condition. HEK293 cells were transfected with 4μg of plasmid vector for 48 hours before lysis. A549 cells were stimulated with 500 U/ml IFN A/D for 24 hours before lysis. In brief, cells were lysed in cytosolic lysis buffer (CLB; 20 mM Tris-HCl pH 7.5, 140 mM NaCl, 5 mM MgCl_2_, 1% Triton-X100) supplemented with 40 U/µl RNasin (Promega), 1 mM dithiothreitol (DTT) and protease inhibitors (cOmplete, Roche) for 30 min. Nuclei and cellular debris was pelleted with centrifugation for 15 minutes at 15000 rpm. Protein concentrations between conditions were normalized using Pierce BCA Protein Assay Kit (Thermo Fisher Scientific) after which input fractions were taken for both western blot and qPCR analysis. To each lysate, 0.5 μg/μl heparin (Sigma) and 0.5 μg/μl sheared salmon sperm DNA (Invitrogen) were added. Pre-clearing was performed for 1 hour with 50 μl unconjugated protein G Dynabeads (Invitrogen) that had been blocked overnight in CLB supplemented with 0.01 mg/ml bovine serum albumin, 0.5 μg/μl yeast tRNA (Ambion), and 0.5 μg/μl sheared salmon sperm DNA. Protocol diverges here based on antibody used in RIP (Supplementary Table. 1). For FLAG RIP, Dynabeads were pre-conjugated overnight with 5μg anti-FLAG (M2, Sigma). After pre-clearing, lysates were incubated with FLAG-conjugated protein G Dynabeads at 4°C for 4 hours with end-over-end rotation. For endogenous RIG-I RIP, pre-cleared lysates were first incubated with 5μg anti-RIG-I antibody (Adipogen) or 5μg mouse IgG1 isotype control at 4°C for overnight with end-over-end rotation. The following day, 50 μl unconjugated blocked protein G Dynabeads were added to each lysate at 4°C for 2 hours with end-over-end rotation. Protocols now converge again. Unbound fraction was taken from each lysate for western blot analysis. Dynabeads were washed 6 times with CLB, switching the tube every second wash to minimize contaminants carryover. A fraction of Dynabeads were kept for western blot analysis, and the rest were resuspended in TRIzol reagent. RNA extraction and cDNA synthesis from RIP and input fraction were isolated as stated above. Enrichment of RNAs of interest was calculated using the ΔΔCt method $\left(2^{\text{-}\Delta \Delta C_{t}}=2^{\text{-}(C_{t,\mathrm{RIP}}\text{-}C_{t,\mathrm{input}}\text{-}\mathrm{lo}g_{2}(20))}\right)$, and normalizing to either the matching empty vector or IgG1 isotype control. In all cases, efficiency of immunoprecipitation was confirmed using Western blot.

### Western blot

Cells were lysed in plates using RIPA buffer (Thermo Fisher Scientific) supplemented with protease inhibitors (cOmplete, Roche) and Genius nuclease (Santa Cruz). Protein concentrations were equalized using the Pierce BCA Protein Assay Kit (Thermo Fisher Scientific). Equalized samples were mixed with bromophenol blue loading dye and 100 mM DTT. Samples were resolved on 4–12% Novex Bis-Tris gels (Invitrogen) and transferred to 0.45 μM PDVF membrane (Sigma) by semi-dry transfer. Membranes were blocked in 5% non-fat dry milk (NFDM) in PBS-T (PBS, 0.1% Tween-20). Membranes were incubated with the indicated primary and secondary antibodies in 5% NFDM in PBS-T and washed 3× 10 minutes in PBS-T after antibody incubation. The RIG-I antibody (Adipogen) was exclusively conjugated to HRP using the FlexAble HRP Antibody Labeling Kit for Mouse IgG1 (Proteintech). Antibodies and dilutions are listed (Supplementary Table. 1). Membranes were developed on an Odyssey CLX-1391 machine (LI-COR Biosciences) or ChemiDoc (Bio-Rad).

### irCLIP

irCLIP was performed as described previously [19], with minor modifications. HEK293 cells expressing 3FLAG-RIG-I variants were UV-crosslinked (254 nm), lysed in NP-40-based buffer, and partially digested with RNase I. 3FLAG-RIG-I-RNA complexes were immunoprecipitated using anti-FLAG-conjugated protein G beads, washed under high-salt conditions, and subjected to 3′ IR adaptor ligation on-bead. Free adaptors were enzymatically removed, and complexes were eluted under denaturing urea conditions and re-immunoprecipitated using anti-FLAG-conjugated protein G beads to increase specificity. Purified complexes were resolved by SDS-PAGE, transferred to nitrocellulose, and RNA-protein bands were excised. RNA was recovered by proteinase K digestion, extracted, and reverse-transcribed using barcoded primers. cDNA was circularized, PCR-amplified, size-selected on TBE gels, and purified to generate sequencing libraries. More detailed description of performed irCLIP protocol available in Supplementary Materials and Methods.

### Alignment and crosslink extraction

Single-end reads were aligned to the GRch38 reference genome using *STAR* [50]. The *Ensembl* gene annotation (v110) was used to extract splice junctions [51]. Soft-clipping was turned off on the 5′ end of the reads (‘*–alignEndsType Extend5pOfRead1’*). Furthermore, we excluded reads with more than 4% mismatched bases (‘*–outFilterMismatchNoverReadLmax 0.04 –outFilterMismatchNmax 999*’) and retained up to 20 alignments per reads in case of multi-mapped reads (‘*–outFilterMultimapNmax 20’*). Chimeric read detection was enabled using ‘*–chimSegmentMin 15 –chimMultimapNmax 15 –chimOutType WithinBAM’*. Finally, we filtered reads with less than 50% of the read length mapped (‘*–outFilterMatchNminOverLread 0.5*’). Ribosomal RNAs (rRNAs) were filtered out in two ways: *SortMeRNA* [52] was used to filter out rRNA before alignment and in downstream analysis, any reads/peaks overlapping rRNA annotations of *GENCODE v47* [53] and *repeatmasker* [54] were removed. After alignment, duplicates were removed using *UMI-tools* [55] with ‘*–method directional’* and using multimapping detection. Deduplicated reads were converted to single-nucleotide crosslinking sites using *BEDTools* [56] by converting read alignment to one nucleotide position upstream of the 5′ end.

### Peak analysis

Peak calling was performed using *PureCLIP* [20]. To reduce memory consumption, the parameters for the HMM were trained on the first three chromosomes (-iv ‘chr1;chr2;chr3;’). To classify peaks into exon, UTR, intron and intergenic classes, the *rtracklayer* package was used in *R* [57, 58]. *FindOverlaps* was used to analyse overlaps between peaks and gene annotations (GENCODE v47 primary assembly). 7SK, 7SL, RNY, Mt_tRNA, Y_RNA, vault_RNA, and ribosome annotations were removed from this assembly before calculating exon, UTR, intron, and intergenic overlap. Subsequently, peaks overlapping intronic and intergenic regions were overlapped with *Pol3Base* and *RepeatMasker* to further subdivide these peaks [21, 54].

### CPM calculation

*FeatureCounts* [59] from the *Subread* software package was used to count reads overlapping RNAPIII annotations (*POL3base*). In case of multi-mapped reads, the *NH* tag assigned by STAR [50] was used to assign a fractional count per alignment. Annotations for tRNA were grouped by amino acid and trinucleotide combination. The other RNAPIII annotations were grouped by the “geneName” feature provided by *POL3base* [21]. Reads that were not assigned to any *POL3base* annotation were subsequently used for another *FeatureCounts* counting overlap with the *Ensembl* genome annotation (v110) [51]. Counts were summed per grouping, which were used to calculate trimmed mean of M values (TMM)-log_2_ normalized counts per million (CPM) per features with *edgeR* [60].

### RNY coverage

A custom *awk* script was used to convert the bed files with crosslinking sites generated previously into a *bedgraph* file, which was converted using the *bedGraphToBigwig* script from UCSC Genome Browser [61]. Again, fractional counts were calculated based on the *NH* tag generated by *STAR* [50]. *Rtracklayer* was used to calculate coverage of crosslinking counts over RNY genes by 10 bp bins [57]. The first bin was calculated centred on the TSS. The other bins were directly connected to this bin in a non-overlapping manner and spanned the whole RNY annotation. Counts were subsequently normalized by the total number of reads per sample (see RPM calculation). A pseudo-count of 1 was added before log_10_ transformation.

### Luciferase reporter assay

Luciferase assays were performed in 96-well format with co-transfection of p125-Firefly Luciferase IFN-β reporter and pRL-TK Renilla reporter. Cells were lysed according to Dual-Luciferase Reporter Assay System (Promega) and firefly and *Renilla* luciferase activity were detected by microplate reader (Tecan).

### Statistical analysis

Unless stated all experiments were repeated a minimum of three times. Data are expressed as the mean ± standard error of the mean. Relevant statistical analyses are described for each individual experiment in the figure legend. Comparisons between more than two groups used ordinary one-way analysis of variance (ANOVA) with Šídák's post-test correction. Appropriate normalization is described in figure legends per each individual experiment. Data were analysed using GraphPad Prism 10 (GraphPad Software, USA). Non-significant (*ns*) ≥ 0.05, *P < 0.05, **P < 0.01, ***P < 0.001, ****P < 0.0001.

## Supplementary Material

kyag013_Supplementary_Data

## Data Availability

The data underlying this article are available in The European Genome-phenome Archive at https://ega-archive.org/studies/EGAS50000001660, and can be accessed with EGAS50000001660.
