# Supplementary material for: An autoinflammatory RIG-I variant causing Singleton-Merten syndrome associates with small non-coding Y-RNAs
Source: Discov Immunol. 2026 Jul 1;5(1):kyag013. doi: 10.1093/discim/kyag013 (PMC13371114; doi:10.1093/discim/kyag013)
Supplement: kyag013_Supplementary_Data [file kyag013_supplementary_data.zip › Supplementary_Legends.docx]

**Supplemental Figure 1 |** **(A)** Annotated representative membrane after irCLIP samples were ran on SDS-PAGE. Arrow indicates the expected molecular weight smear comprised of 3F-RIG-I bound to associated RNA and 3’ IR-adaptor. **(B)** Annotated representative agarose gel after irCLIP libraries were size separated. Arrow indicates the cDNA species between 150-500nt which were excised prior to sequencing. Adaptor and primer artefacts labelled. **(C)** JBrowse2 tracks of irCLIP read coverage across *VTRNA1* locus in RIG-I^WT^ and RIG-I^C268F^ replicates. **(D)** JBrowse2 tracks of irCLIP read coverage across 2 representative *tRNA-Pro* loci in RIG-I^WT^ and RIG-I^C268F^ replicates. **(E)** Crosslinking site analysis showing irCLIP read density across *VTRNA* RNAs. **(F)** Crosslinking site analysis showing irCLIP read density across tRNA^His^ family.

**Supplemental Figure 2 |** **(A)** Native RIP-qPCR analysis of *VTRNA1* transcripts, *tRNA-Lys-AAA*, *and tRNA-Asn-AA*C RNAs bound to RIG-I. Data is expressed as a fraction of input RNA levels. Enrichment of these RNAs was then calculated by normalisation to the empty vector (EV) control. Statistical significance was assessed by one-way ANOVA on log_10_‑transformed data. *n*=3. **(B)** Western blot of formaldehyde RIP after FLAG-IP performed on lysates from HEK293 cells overexpressing 3F-RIG-I (WT/C268F/E373A/T347A) for 48 hours. *n*=1. **(C)** Formaldehyde RIP-qPCR analysis RNAs bound to RIG-I. Data is expressed as a fraction of input RNA levels. Enrichment of these RNAs was then calculated by normalisation to the empty vector (EV) control. *n*=1. **(D)** Strategy of CRISPR-editing MAVS^KO^ into A549 cells. Sequencing chromatograms from selected clone showed the introduction of a premature stop codon (*MAVS^L84Rfs*32^*). **(E)** Validation of MAVS^KO^ line by transfection with low-molecular weight poly(I:C) (500ng) for 7 hours. RNA was isolated for qPCR analysis of *IFNL1* expression. *n*=1. **(F)** Western blot showing both MAVS expression in A549 wild-type MAVS cells compared to MAVS^KO^ lines and RIG-I expression in two RIG-I^WT^ and two RIG-I^C268F^ clones. **(G)** Endogenous RIP-qPCR analysis of *VTRNA1-1*, *VTRNA1-2*, and *tRNA-Pro-TGG* RNA bound to RIG-I. Data is expressed as a fraction of input transcript levels. Enrichment of targets was then calculated by normalisation to the RIG-I^WT^ #1 IgG control. Statistical significance was assessed by one-way ANOVA on log_10_‑transformed RIG‑I IP data. *n*=3. **(H)** Endogenous RIP-qPCR analysis of *SNAR-A* RNA bound to RIG-I. Data is expressed as a fraction of input transcript levels. Enrichment of target was then calculated by normalisation to the RIG-I^WT^ #1 IgG control. *n*=1. All data (unless stated otherwise) are represented as means ± SEM. (* *P*<0.05, ** *P*<0.01, *** *P*<0.001, **** *P*<0.0001).
