# Supplementary material for: An autoinflammatory RIG-I variant causing Singleton-Merten syndrome associates with small non-coding Y-RNAs
Source: Discov Immunol. 2026 Jul 1;5(1):kyag013. doi: 10.1093/discim/kyag013 (PMC13371114; doi:10.1093/discim/kyag013)
Supplement: kyag013_Supplementary_Data [file kyag013_supplementary_data.zip › Thompson et al. RIG-I C268F in SMS - Supplementary Materials & Methods.docx]

**Supplementary Materials and Methods**

**Supplementary irCLIP method**

*Cell culture and UV-crosslinking.* HEK293 cells were plated at a density of 7x10^6^ cells in 15cm culture dishes in which one culture dish was used per irCLIP condition. On the following day HEK293 cells were transfected with 8μg of pcDNA3.1 containing either empty vector, 3FLAG-RIG-I^WT^, or 3FLAG-RIG-I^C268F^ for 48 hours. On the day of harvest, cells were washed with 3mL ice-cold PBS and crosslinked with 150mJ/cm^2^ UV-irradiation (254 nm), using a AnalytikJena CL-1000 UV crosslinker. After irradiation, cells were scraped, pelleted, and snap-frozen.

*Cell lysis and partial RNA digestion.* Cell-pellet was lysed in 1mL irCLIP cytosolic lysis buffer (iCLB) (20mM Tris-HCl, pH7.4, 140mM NaCl, 5mM MgCl_2_, 1% Triton X-100, supplemented with 1mM DTT, protease inhibitors, and 4U Turbo DNase (Invitrogen)) for 30 minutes, followed by 10 minute centrifugation at 15000rpm to remove nuclei and cellular debris. Protein concentrations were equalised by BCA, after which input fractions were taken for both western blot and total RNA-seq analysis. Partial RNA digestion was performed using 5U/mg/mL of RNase I (Thermo Scientific, EN0602) along with 4U Turbo DNase per sample and was incubated at 37^o^C for 3 minutes plus shaking at 1100rpm, followed by cooling on ice for 5 minutes. Samples were centrifuged at 4^o^C for 10 minutes at 15000rpm and transferred to a new tube.

*Immunoprecipitation.* For immunoprecipitation, 100μL protein G Dynabeads (Invitrogen) were washed with iCLB and incubated overnight with 10μg anti-FLAG (M2, Sigma). Conjugated Dynabeads were added to RNase-digested lysates and incubated overnight at 4^o^C with end-to-end rotation. The following day, unbound fraction was taken from each sample for western blot analysis. Dynabeads were washed twice with high-salt wash buffer (HS-WB) (50mM Tris-HCl, pH7.4, 1M NaCl, 1mM EDTA, 1% Igepal CA-630 (Sigma I8896), 0.1% SDS, 0.5% sodium deoxycholate) with 1 minute 4^o^C rotation between each wash. In order to prevent salt/RNase/DNase cross-over, Dynabeads were washed in PNK Wash Buffer (PNK-WB) (20mM Tris-HCl pH7.4, 10mM MgCl_2_, 0.2% Tween-20), transferred to a new tube, and washed a second time in PNK-WB.

*3’ RNA dephosphorylation.* For 3’ RNA dephosphorylation, Dynabeads were resuspended in 40uL of the following buffer: (8μL 5x PNK pH6.5 buffer, 1μL PNK (NEB M0201L), 0.5µl FastAP alkaline phosphatase (Thermo Fisher Scientific, EF0654), 0.5µL RNasin Plus (Promega N261B), 30µl water). 5x PNK pH6.5 buffer is comprised of the following and stored at -20^o^C (350mM Tris-HCl pH6.5, 50mM MgCl_2_, 5mM DTT). This mix was incubated in a thermomixer for 40 minutes at 37^o^C at 1100rpm.

*3’ adaptor ligation.* Supernatant was discarded from Dynabeads and beads were washed with 1x DDT-free ligation buffer (50mM Tris-HCL pH7.5, 10mM MgCl_2_). Dynabeads were then resuspended in 25μL of 3’ adaptor ligation mix (6.3µl water, 3µl 10x ligation buffer (no DTT), 0.8µl 100% DMSO, 2.5µl T4 RNA ligase I - high concentration (M0437M NEB), 0.4µl RNasin, 0.5µl PNK (NEB M0201L), 2.5µl 3′ IR adaptor (stock 1µM), 9µl 50% PEG8000). 3’ IR adaptor sequence is listed (Table. 5). This mix was incubated for 75 minutes at room temperature with gentle mixing by flicking every 10 minutes. Dynabeads were washed twice with HS-WB with rotation. Dynabeads were washed once with PNK-WB.

*Free adaptor removal.* Dynabeads were resuspended in 1mL of PNK-WB and transferred to a new tube. Supernatant was discarded and 20μL of adaptor removal buffer was added to each sample (12.5µl water, 2µl NEB Buffer 2, 0.5µl 5’ Deadenylase (NEB M0331S), 0.5µl RecJf endonuclease (NEB M0264S), 0.5µl RNasin, 4µl PEG400). This mix was incubated in a thermomixer for 1 hour at 30^o^C and then 30 minutes at 37^o^C at 1100rpm shaking. Dynabeads were washed twice with HS-WB with rotation. Dynabeads were washed once with PNK-WB.

*Urea extraction and re-immunoprecipitation.* Dynabeads were incubated in a thermomixer with 80μL of 1.25x urea cracking buffer (66.6mM Tris-HCl pH7.4, 8M urea, 1.33% SDS) at 65^o^C for 3 minutes at 1100rpm. Supernatant was collected off Dynabeads and 920μL of Tween-20 IP buffer (50mM Tris-HCl pH7.4, 150mM NaCl, 0.5% Tween-20, 0.1mM EDTA) was added to supernatant. For re-immunoprecipitation, elution was added to 100μL protein G Dynabeads pre-coupled to 10μg anti-FLAG and incubated overnight at 4^o^C with end-to-end rotation. Dynabeads were washed twice with HS-WB with rotation. Dynabeads were washed once with PNK-WB.

*SDS-PAGE and nitrocellulose transfer.* Dynabeads were incubated with 20μL pre-heated (70^o^C) Laemmli loading buffer with 100mM DTT for 1 minute at 70^o^C with shaking. Samples were resolved on 4-12% NuPAGE Bis-Tris gels (Invitrogen), transferred on to nitrocellulose membrane (BioRad), and developed on an Odyssey CLX-1391 machine (LI-COR Biosciences).

*RNA isolation.* Smears above 110kDa were cut from nitrocellulose membrane and mixed with 10μL Proteinase K (Roche) and 190μL PK/SDS buffer (10 mM Tris-HCl, pH7.4, 100mM NaCl, 1mM EDTA, 0.2% SDS) for 1 hour at 50^o^C with 1100rpm shaking. Solution was mixed with 200μL phenol:chloroform:isoamyl alcohol (Sigma) and added to Phase Lock Gel Heavy tubes (VWR) followed by 5 minute centrifugation at 13000rpm. 800μL chloroform was added to top of tubes followed by 5 minute centrifugation at 13000rpm. Aqueous phase was transferred to new tube and precipitated with 0.75μL Glycoblue, 20μL 5M NaCl, and 500μL 100% ethanol and frozen overnight. Samples were centrifuged at 15000rpm for 30 minutes, and pellet was washed with 80% ethanol and re-centrifuged. RNA pellet was resuspended in 5.5μL water.

*Reverse transcription.* Individual samples were barcoded by mixing with 1μL of 1pmol/μL reverse transcription oligos (Table. 5) and 0.5μL 10mM dNTP and heated to 65^o^C for 5 minutes. To this mix was added 0.25μL SuperScript IV (Invitrogen), 2μL 5x SuperScript IV buffer (Invitrogen), 0.5μL 0.1M DTT, and 0.25μL RNasin. Mix was incubated with the following protocol: 5 minutes at 25^o^C, 5 minutes at 50^o^C, 5 minutes at 55^o^C. After incubation, 1.25μL of 1M NaOH was added and incubated at 85^o^C for 15 minutes followed by 1.25μL of 1M HCl.

*cDNA purification and circularisation.* cDNA was purified using AMPure XP beads (Agencourt) according to manufacturer’s protocol and eluted in 9μL water. For cDNA circularisation, purified cDNA was added to CircLigase II mix was added (0.75μL CircLigase II (Epicentre), 1.5μL 10x CircLigase II Buffer (Epicentre), 0.75μL 50mM MnCl_2_, 3μL 5M betaine) and incubated at 60^o^C for 2 hours. Circularised cDNA was purified again with AMPure XP beads and eluted in 10μL water.

*Library amplification.* cDNA library was amplified using P5/P3 Solexa primers (Table. 5) and Phusion High-Fidelity PCR Master Mix (Thermo Fisher Scientific) with 17 amplification cycles. PCR product was run on 6% TBE gels (Invitrogen) and bands in the range of 175-500 nucleotides were exercised. Gel was macerated and incubated in crush-soak buffer (500mM NaCl, 1mM EDTA, 0.05% SDS) at 65^o^C for 2 hours. Supernatant was transferred to Costar SpinX columns (Corning) and centrifuged at 13000rpm for 1 minute. DNA library was precipitated from flow-through with GlycoBlue, sodium acetate and ethanol.

*Sequencing.* irCLIP cDNA library was subject to single-end total RNA sequencing by Utrecht Sequencing Facility (University Medical Center Utrecht).
