# Supplementary material for: An autoinflammatory RIG-I variant causing Singleton-Merten syndrome associates with small non-coding Y-RNAs
Source: Discov Immunol. 2026 Jul 1;5(1):kyag013. doi: 10.1093/discim/kyag013 (PMC13371114; doi:10.1093/discim/kyag013)
Supplement: kyag013_Supplementary_Data [file kyag013_supplementary_data.zip › Thompson et al. RIG-I C268F in SMS - Supplementary Tables.docx]

**Supplementary Table 1 | Antibodies**

| **Antibody target** | **Application** | **Host species** | **Working dilution** | **Source** | **Code/Clone** | **Conjugated** |
| --- | --- | --- | --- | --- | --- | --- |
| RIG-I | WB | Mm | 1:1000 | Adipogen | AG-20B-0009-C100 | - |
|  | RIP | Mm | 5μg/mL | Adipogen | AG-20B-0009-C100 | - |
| FLAG | WB | Mm | 1:10000 | Merck | F3165/M2 | HRP |
|  | RIP | Mm | 5μg/IP | Merck | F3165/M2 | - |
|  | irCLIP | Mm | 5μg/IP | Merck | F3165/M2 | - |
| MAVS | WB | Mm | 1:500 | SCB | E-3 | - |
| ISG60 | WB | Mm | 1:1000 | SCB | B-7 | - |
| RSAD2 | WB | Rb | 1:1000 | CST | 13996 | - |
| ACTB | WB | Rb | 1:1000 | SCB | sc-50509 | HRP |
| Anti-Mouse | WB | Go | 1:10000 | Invitrogen | G-21040 | HRP |
| Anti-Rabbit | WB | Go | 1:10000 | SB | 4050-05 | HRP |
| Control IgG1 | RIP | Mm | 5μg/IP | SCB | sc-2025 |  |

**WB** – Western blot, **IP** – Immunoprecipitation, **RIP** – RNA immunoprecipitation

**Mm** – Mouse, **Rb** – Rabbit, **Go** – Goat

**SCB** – Santa Cruz Biotechnology Inc USA, **CST** – Cell Signalling Technology USA , **SB** – Southern Biotech

**Supplementary Table 2 | qPCR oligos**

| **Target** | **Primer sequences used for qPCR** | |
| --- | --- | --- |
|  | **Sequence (5’-3’)** | |
| *VTRNA1-1* | F | TTAGCTCAGCGGTTACTTCGACAGTTC |
|  | R | AAAAGGACTGGAGAGCGCCC |
| *VTRNA1-2* | F | GGCTGGCTTTAGCTCAGCGG |
|  | R | AAAAGAGCTGGAAAGCACCC |
| *VTRNA1-3* | F | AGCGGTTACTTCGCGTGTCATC |
|  | R | AAGAGGGCTGGAGAGCGCC |
| *RNY1* | F | GGCTGGTCCGAAGGTAGTGAG |
|  | R | GGGGGAAAGAGTAGAACAAGG |
| *RNY3* | F | CCGAGTGCAGTGGTGTTTAC |
|  | R | AAGCAGTGGGAGTGGAGAA |
| *RNY4* | F | TCCGATGGTAGTGGGTTATCA |
|  | R | AAAGCCAGTCAAATTTAGCAGT |
| *RNY5* | F | AGTTGGTCCGAGTGTTGTGG |
|  | R | AACAGCAAGCTAGTCAAGCG |
| *ACTB* | F | CACTCTTCCAGCCTTCCTTC |
|  | R | TACAGGTCTTTGCGGATGTC |
| *RSAD2* | F | AGCAATGGAAGCCTGATCC |
|  | R | ACTTCCTCGTCAAAGCTGTC |
| *IFIT1* | F | CAGAATAGCCAGATCTCAGAGG |
|  | R | CCAGACTATCCTTGACCTGATG |
| *IFNB1* | F | AGTAGGCGACACTGTTCGTG |
|  | R | GTCTCATTCCAGCCAGTGCT |
| *RO60* | F | ATGTGCATGGTTGTCACACG |
|  | R | CACCTGCTGGGATCTGACTC |
| *RNA18S* | F | GTAACCCGTTGAACCCCATT |
|  | R | CCATCCAATCGGTAGTAGCG |
| *RIG-I* | F | CGGAAGACCCTGGACCCTAC |
|  | R | ACTGCACCTCTTCCTCCCTAA |
| *tRNA-Asn-AAC* | F | TCTCTGTGGCGCAATCGGTT |
|  | R | CGTCCCTGGGTGGGCTCGAA |
| *tRNA-Lys-AAA* | F | CGCCTGGACAGGGACTTGAA |
|  | R | CCTGGGTAGCTCAGTCGGTA |
| *tRNA-Pro-TGG* | F | GGCTCGTTGGTCTAGGGGTATG |
|  | R | GGGCTCGTCCGGGATTTGAAC |
| *IFNL1* | F | GTGACTTTGGTGCTAGGCTTG |
|  | R | GCCTCAGGTCCCAATTCCC |
| *SNAR-A* | F | GGAGCCATTGTGGCTCAG |
|  | R | ACCCATGTGGACCAGGCT |

**Supplementary Table 3 | sgRNA oligos and HDR template oligo**

| **Target** | **Purpose** | **crRNA sequences used for CRISPR-Cas9 gene editing** |
| --- | --- | --- |
|  |  | **Sequence (5’-3’)** |
| *MAVS* | Deletion | CTGTGAGCTAGTTGATCTCG |
| *RIG-I* | Homology Directed Repair | CCTTACATGTTAAAAGGTTG |
| **Target** | **Purpose** | **HDR template sequences used for CRISPR-Cas9 HDR** |
|  |  | **Sequence (5’-3’)** |
| *RIG-I* | c.801T>C  p.Gly266Gly  c.803G>T  p.Cys268Phe | ATGAGTTACATGTTTTCTTTTTTCCTTACATGTTAAAAGG**C**T**T**TG  GAAAAACCTTTGTTTCACTGCTTATATGTGAACATCATC |

**Supplementary Table 4 | PCR Sequencing oligos**

| **Target** | **Primer sequences used for qPCR** | |
| --- | --- | --- |
|  | **Sequence (5’-3’)** | |
| *MAVS (for CRISPR)* | F | CATACCTGGCCCTGTCCTG |
|  | R | AGCCGTGAAAGTTAGGGGAG |
| *RIG-I (for CRISPR)* | F | CTTTTGGGTCCTGGCTGTTG |
|  | R | CCATGAAGGGATAACATCTGGA |

**Supplementary Table 5 | irCLIP Oligos**

*Infrared adaptor, reverse transcription oligos, and library PCR oligos*

| **Name** | | **Sequence (5’-3’)** |
| --- | --- | --- |
| 3′ IR adaptor | | /5Phos/AG ATC GGA AGA GCG GTT CAG AAA AAA AAA AAA /iAzideN/AA AAA AAA AAA A/3Bio/ |
| **Used with sample #** | **Name** | **Sequence (5’-3’)** |
| Repeat 1 – EV | irCLIP_ddRT_12 | /5Phos/ WWW GTGGA NNNN AGATCGGAAGAGCGTCGTGAT /iSp18/ GGATCC /iSp18/ TACTGAACCGC |
| Repeat 1 – RIG-I^WT^ | irCLIP_ddRT_13 | /5Phos/ WWW TCCGG NNNN AGATCGGAAGAGCGTCGTGAT /iSp18/ GGATCC /iSp18/ TACTGAACCGC |
| Repeat 1 – RIG-I^C268F^ | irCLIP_ddRT_16 | /5Phos/ WWW TTAAA NNNN AGATCGGAAGAGCGTCGTGAT /iSp18/ GGATCC /iSp18/ TACTGAACCGC |
| Repeat 2 – EV | irCLIP_ddRT_17 | /5Phos/ WWW AAATG NNNN AGATCGGAAGAGCGTCGTGAT /iSp18/ GGATCC /iSp18/ TACTGAACCGC |
| Repeat 2 – RIG-I^WT^ | irCLIP_ddRT_18 | /5Phos/ WWW AAGGT NNNN AGATCGGAAGAGCGTCGTGAT /iSp18/ GGATCC /iSp18/ TACTGAACCGC |
| Repeat 2 – RIG-I^C268F^ | irCLIP_ddRT_19 | /5Phos/ WWW AATAC NNNN AGATCGGAAGAGCGTCGTGAT /iSp18/ GGATCC /iSp18/ TACTGAACCGC |
| Repeat 3 – EV | irCLIP_ddRT_20 | /5Phos/ WWW ACGCA NNNN AGATCGGAAGAGCGTCGTGAT /iSp18/ GGATCC /iSp18/ TACTGAACCGC |
| Repeat 3 – RIG-I^WT^ | irCLIP_ddRT_21 | /5Phos/ WWW ACTTG NNNN AGATCGGAAGAGCGTCGTGAT /iSp18/ GGATCC /iSp18/ TACTGAACCGC |
| Repeat 3 – RIG-I^C268F^ | irCLIP_ddRT_22 | /5Phos/ WWW AGAGC NNNN AGATCGGAAGAGCGTCGTGAT /iSp18/ GGATCC /iSp18/ TACTGAACCGC |
| **Name** | **Sequence (5’-3’)** | |
| P5 Solexa | AATGATACGGCGACCACCGAGATCTACACTCTTTCCCTACACGACGCTCTTCCGATCT | |
| P3 Solexa | CAAGCAGAAGACGGCATACGAGATCGGTCTCGGCATTCCTGCTGAACCGCTCTTCCGATCT | |

**Supplementary Table 6 | Site-directed mutagenesis oligos**

| **Target** | **Purpose** | **Sequences used for site-directed mutagenesis** | |
| --- | --- | --- | --- |
|  |  | **Sequence (5’-3’)** | |
| *RIG-I* | c.803G>T  p.Cys268Phe | F | CACAATAATATGTGCTCCTACAGGTTTTGGAAAAACCTTTGTTTCA |
|  |  | R | TGAAACAAAGGTTTTTCCAAAACCTGTAGGAGCACATATTATTGTG |
|  | c.1039A>G  p.Thr347Ala | F | ATTGTTGAGAACAATGACATCATCATTTTAGCTCCACAGATTCTTGT |
|  |  | R | ACAAGAATCTGTGGAGCTAAAATGATGATGTCATTGTTCTCAACAAT |
|  | c.1117G>C  p.Glu393Gln | F | ATCCATCTTTACTTTGATGATATTTGATCAATGCCACAACACTAGTAA |
|  |  | R | TTACTAGTGTTGTGGCATTGATCAAATATCATCAAAGTAAAGATGGAT |
|  | c.1118A>C  p.Glu393Ala | F | CTTTACTTTGATGATATTTGATGCATGCCACAACACTAGTAAACAAC |
|  |  | R | GTTGTTTACTAGTGTTGTGGCATGCATCAAATATCATCAAAGTAAAG |
