# Supplementary figures and images for: An autoinflammatory RIG-I variant causing Singleton-Merten syndrome associates with small non-coding Y-RNAs
Source: Discov Immunol. 2026 Jul 1;5(1):kyag013. doi: 10.1093/discim/kyag013 (PMC13371114; doi:10.1093/discim/kyag013)

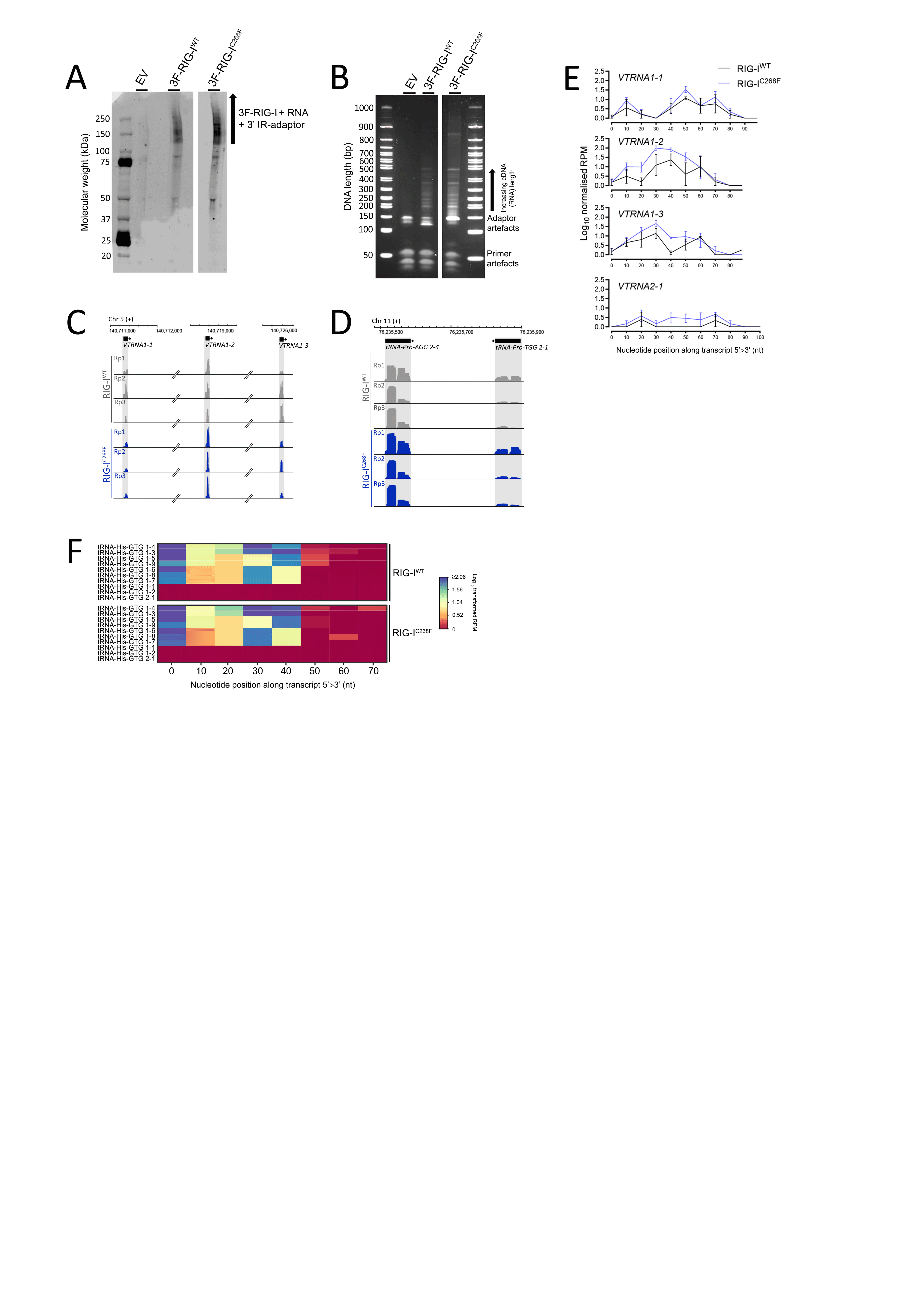

Supplement: kyag013_Supplementary_Data [file kyag013_supplementary_data.zip › Supplemental Figure 1.gif]

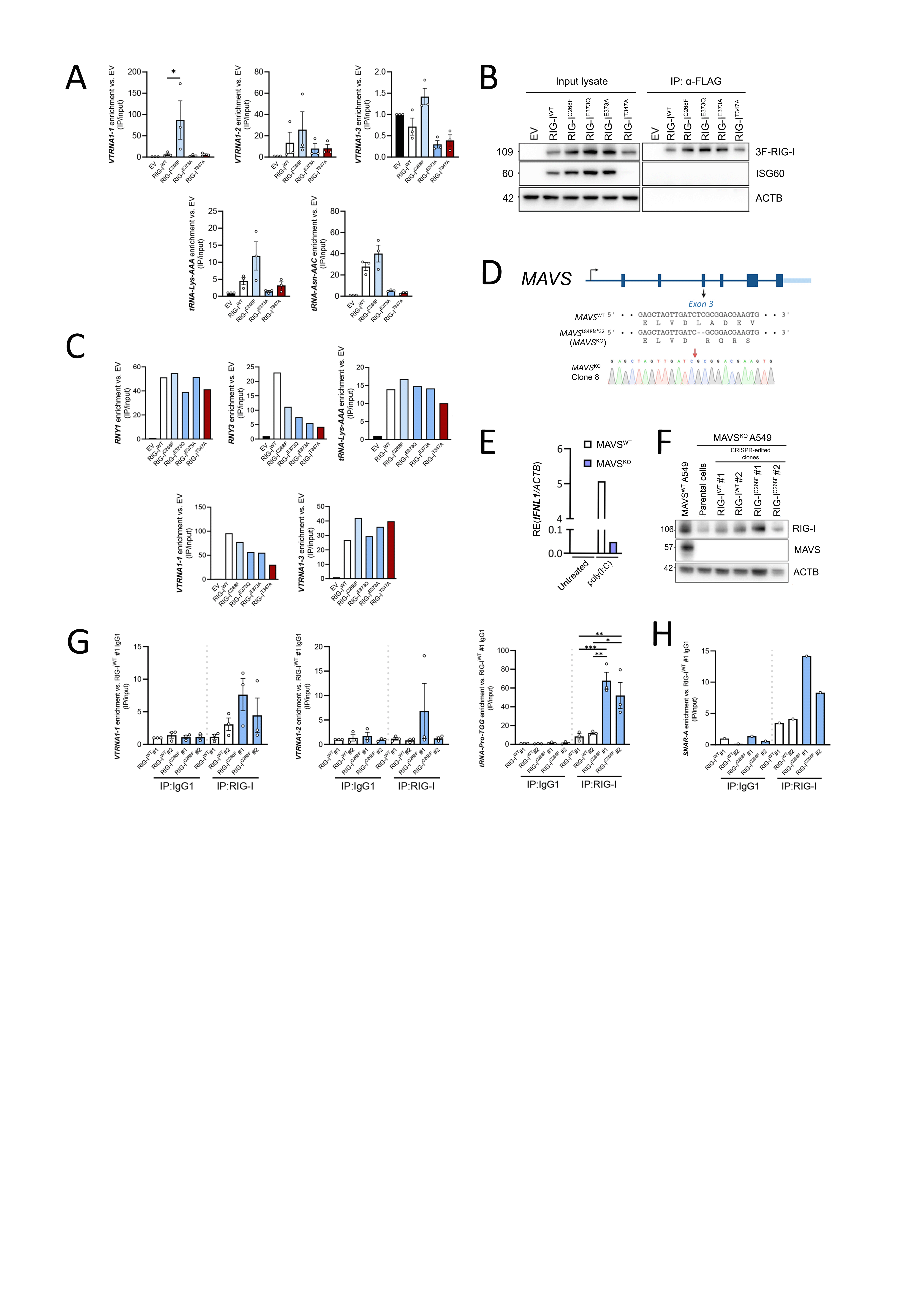

Supplement: kyag013_Supplementary_Data [file kyag013_supplementary_data.zip › Supplemental Figure 2.gif]
